# Bipolar disorder type II - will the new classification help in setting an adequate diagnosis

**DOI:** 10.1192/j.eurpsy.2023.1485

**Published:** 2023-07-19

**Authors:** S. Vuk Pisk, E. Ivezic, L. Senjug Mance, K. Matic, D. Svetinovic, V. Grosic, I. Filipcic

**Affiliations:** 1 Psychiatric Clinic Sveti Ivan, Zagreb; 2 Faculty of Dental Medicine and Health, Josip Juraj Strossmayer University of Osijek, Osijek; 3 School of Medicine, University of Zagreb, Zagreb, Croatia

## Abstract

**Introduction:**

Bipolar affective disorder type II is often misunderstood, neglected and rarely receives the attention it deserves and often remains undiagnosed. Despite its neglect and insufficient diagnosis, it is an important diagnostic entity because it causes significant suffering and functional impairment, a chronic course of the disease and a high suicide rate. Cognitive impairments and multiple comorbidities that significantly affect the course and outcome of the disease are common.

**Objectives:**

The purpose of this research was to determine the extent of the deficiency in diagnosing bipolar affective disorder type II in daily clinical work.

**Methods:**

A total of 82 adult psychiatric inpatients diagnosed with affective disorders (depressive disorders and bipolar affective disorders) and borderline disorders participated in this study. They completed HCL-32, MDQ and PHQ-9 questionairres. The average age of the sample is 43.9 years. A total of 76.8% were women in the sample.

**Results:**

72.8% of respondents achieved a score above 14 on the HCL-32 questionnaire and thus met the criteria for possible hypomania. All three criteria for mania on the MDQ questionnaire were satisfied by 27.5% of respondents. 68% of respondents have moderate or severe symptoms of depression according to PHQ-9.

**Conclusions:**

The results confirmed our assumption about the lack of recognition and diagnosis of bipolar affective disorder type II. Only 5 respondents (6.1%) were diagnosed with BAP II upon arrival. After the research, 73% of them met the criteria for diagnosing BAP II. As a correctly established diagnosis affects the selection of adequate therapy, we have tried to emphasize the importance of correct recognition of BAP II.

**Disclosure of Interest:**

None Declared

